# *Fasciola hepatica* Immune Regulates CD11c^+^ Cells by Interacting with the Macrophage Gal/GalNAc Lectin

**DOI:** 10.3389/fimmu.2017.00264

**Published:** 2017-03-15

**Authors:** Ernesto Rodríguez, Paula Carasi, Sofía Frigerio, Valeria da Costa, Sandra van Vliet, Verónica Noya, Natalie Brossard, Yvette van Kooyk, Juan J. García-Vallejo, Teresa Freire

**Affiliations:** ^1^Grupo de Inmunomodulación y Desarrollo de Vacunas, Departamento de Inmunobiología, Facultad de Medicina, Universidad de La República, Montevideo, Uruguay; ^2^Department of Molecular Cell Biology and Immunology, VU University Medical Center, Amsterdam, Netherlands

**Keywords:** helminth, dendritic cell, C-type lectin receptors, glycans, macrophage Gal/GalNAc lectin, immune regulation

## Abstract

Fasciolosis, caused by *Fasciola hepatica* and *Fasciola gigantica*, is a trematode zoonosis of interest in public health and livestock production. Like other helminths, *F. hepatica* modulates the host immune response by inducing potent polarized Th2 and regulatory T cell immune responses and by downregulating the production of Th1 cytokines. In this work, we show that *F. hepatica* glycans increase Th2 immune responses by immunomodulating TLR-induced maturation and function of dendritic cells (DCs). This process was mediated by the macrophage Gal/GalNAc lectin (MGL) expressed on DCs, which recognizes the Tn antigen (GalNAc-Ser/Thr) on parasite components. More interestingly, we identified MGL-expressing CD11c^+^ cells in infected animals and showed that these cells are recruited both to the peritoneum and the liver upon *F. hepatica* infection. These cells express the regulatory cytokines IL-10, TNFα and TGFβ and a variety of regulatory markers. Furthermore, MGL^+^ CD11c^+^ cells expand parasite-specific Th2/regulatory cells and suppress Th1 polarization. The results presented here suggest a potential role of MGL in the immunomodulation of DCs induced by *F. hepatica* and contribute to a better understanding of the molecular and immunoregulatory mechanisms induced by this parasite.

## Introduction

*Fasciola hepatica* is a worldwide-distributed parasitic flatworm that causes fasciolosis, a zoonotic disease that affects mainly livestock and causes significant economic losses worldwide (1). In addition, the World Health Organization (WHO) estimates that around 2.5 million people are infected around the world and several millions are at risk (1). Like other helminths, *F. hepatica* modulates the host immune response by inducing potent polarized Th2 and regulatory T cell immune responses and by downregulating the production of Th1 cytokines (2–5). This immunoregulated environment favors the differentiation of regulatory T cells (3), the alternative activation of macrophages (5), and the modulation of the activity of both dendritic cells (DCs) and mast cells (2, 6–8). Helminths express carbohydrate-containing glycoconjugates on their surface and they release glycan-rich excretion/secretion products that can be very important in their life cycles and pathology, since they can participate in immune escape (9). In this context, we have recently described that glycans structures produced by *F. hepatica* participate in the modulation of DC maturation and mediate the production of IL-10 and IL-4 during infection (10).

Parasite glycans are recognized by the immune system through the interaction of C-type lectin receptors (CLRs), a large family of calcium-dependent glycan-binding proteins that present structural homology in their carbohydrate recognition domain (11). Several reports have highlighted the role of CLRs in mediating the internalization of parasite glycoconjugates and cell-surface signaling, leading to a modulation of the host immune response (12–14). Macrophage Gal/GalNAc lectin (MGL), also known as CLEC4A or CD301, is a type II transmembrane protein expressed on professional antigen-presenting cells (15, 16). MGL displays a remarkable specificity for terminal *N-*acetyl-galactosamine (GalNAc) moieties, including the Tn antigen (αGalNAc-O-Ser/Thr) and LacDiNAc (GalNAcβ1-4GlcNAc, LDN). While there is only one MGL in humans (hMGL), two orthologs are present in mice, mMGL1 (CD301a) and mMGL2 (CD301b), which differ in their glycan specificity (17, 18). Interestingly, although mMGL1 is more structurally similar to hMGL, mMGL2 and hMGL display similar ligand specificity (19). In contrast, mMGL1 recognizes the Lewis^x^ [Galβ1–4(αFuc1–3)GlcNAc] and Lewis^a^ [Galβ1–3(αFuc1–4)GlcNAc] antigens. Several reports have demonstrated that both human and murine MGL can recognize glycoconjugates present in helminth parasites, such as *Schistosoma mansoni* (20), *Trichuris suis* (21), and *Taenia crassiceps* (22). Furthermore, it has been proposed that MGL2^+^ dermal DCs are specialized in the induction of Th2 responses both in allergy and helminth-infection models (22).

Given that *F. hepatica* glycans modulate DC maturation inducing a Th2/regulatory-polarized immune response (2–5) and our group has previously identified the Tn antigen expressed on *F. hepatica* glycoconjugates (23), the simplest mucin type *O*-glycan structure composed of *N*-acetyl-d-galactosamine with a glycosidic α-linkage to serine/threonine residues in glycoproteins (17, 23), we set out to evaluate the potential role of MGL in the recognition of parasite glycans as well as a mediator of *F. hepatica*-induced immunoregulation.

Our results indicate that the Tn antigen expressed by *F. hepatica* can modulate the TLR2-induced maturation of human monocyte-derived DCs (mo-DCs) in a process mediated by hMGL by upregulating the production of IL-10 and TNFα. Furthermore, we show that mMGL2^+^ CD11c^+^ F4/80^lo^ cells are recruited to the peritoneum of infected mice. Interestingly, these cells express the regulatory cytokines IL-10, TNFα, and TGFβ and a variety of regulatory markers. The results presented here constitute the first report about the participation of mMGL2^+^ CD11c^+^ in the expansion of Th2/regulatory-immune responses and in the suppression of Th1 polarization during an helminth infection, suggesting a potential role of MGL in the immunomodulation induced by *F. hepatica* and contribute to a better understanding of the molecular and immunoregulatory mechanisms induced by this parasite.

## Materials and Methods

### Ethics Statement

Mouse experiments were carried out in accordance with strict guidelines from the National Committee on Animal Research (Comisión Nacional de Experimentación Animal, CNEA, http://www.cnea.org.uy, National Law 18.611, Uruguay) according to the international statements on animal use in biomedical research from the Pan American Health Organization and WHO. Adult worms were collected from bovine livers during the routine work of a local abattoir (Frigorífico Carrasco) in Montevideo (Uruguay). Protocols were approved by the Uruguayan Committee on Animal Research (Comisión Honoraria de Experimentación Animal, CHEA Protocol Numbers: 071140-001822-11 and 071140-000143-12).

### Mice

Six- to eight-week-old female BALB/c mice were obtained from DILAVE Laboratories (Uruguay). Animals were kept in the animal house (URBE, Facultad de Medicina, UdelaR, Uruguay) with water and food supplied *ad libitum*, mouse handling and experiments were carried out in accordance with strict guidelines from the National Committee on Animal Research (Comisión Nacional de Experimentación Animal, CNEA, National Law 18.611, Uruguay). Adult worms were collected during the routine work of a local abattoir (Frigorífico Carrasco) in Montevideo (Uruguay). All procedures involving animals were approved by the Universidad de la República’s Committee on Animal Research (Comisión Honoraria de Experimentación Animal, CHEA Protocol Numbers: 071140-001822-11 and 071140-000143-12).

### Preparation of Protein Lysates from *F. hepatica*

Live adult worms of *F*. *hepatica* were obtained from the bile ducts of bovine livers, washed in phosphate-buffered saline (PBS) pH 7.4, then mechanically disrupted and sonicated. After centrifugation at 40,000 × *g* for 60 min, supernatants were collected and dialyzed against PBS. The obtained lysate (FhTE) was quantified and stored at −80°C. The endotoxin levels were determined by using the Limulus Amebocyte Lysate kit Pyrochrome (Associates of Cape Cod). Protein preparations showed very low levels of endotoxins and were not able to induce DC maturation on their own. The concentration of all *F. hepatica* extracts used in culture experiments did not induce signaling through TLR4 or TLR2 nor modify cell viability of moDCs evaluated by flow cytometry, as shown in Figure S1 in Supplementary Material. For a tegumental extract of *F. hepatica*, adult worms were incubated in 1% deoxycholic acid in 0.15 M glycine (pH 9.0), 0.5 M NaCl for 60 min at 37°C. The deoxycholate extracted material was centrifuged at 20,000 × g for 60 min, dialyzed against PBS, and stored at −80°C until used.

### Cells

Monocytes were isolated from peripheral blood mononuclear cells from buffy coats of healthy human donors (Sanquin, The Netherlands) by a lymphoprep gradient (Axis-Shield, San Diego, CA, USA) and subsequent percoll gradient centrifugation (GE Healthcare Life Science, Netherlands). Informed consent was obtained from all blood donors. DCs were generated by culturing purified monocytes in complete medium consisting of RPMI 1,640 (Thermo Fisher Scientific, Netherlands) supplemented with 10% fetal bovine serum (BioWhittaker), 1,000 U/ml penicillin/streptomycin (Lonza, Netherlands), and 2 mM glutamine (Lonza, Netherlands) in combination with IL-4 (262.5 U/ml; Biosource, Belgium) and GM-CSF (112.5 U/ml; Biosource, Belgium) for 4–5 days. After that time, cells were harvested and MGL expression was confirmed by flow cytometry using a specific hMGL antibody (1G6.6) (24). For DC-maturation assays, mo-DCs (2 × 10^5^/well) were incubated at 37°C and 5% CO_2_ in 96-well plates with plate-bound FhTE (125 μg/ml) in the presence or absence of Pam3CysK4 (TLR1/2, 10 μg/ml) or LPS (TLR4, 10 ng/ml). When appropriate, DCs were preincubated for 60 min at 37°C with the blocking anti-MGL antibody (1G6.6). IL-6, IL-10, and TNFα levels were determined by specific ELISAs (eBiosciences, CA or BioSource, Belgium) after overnight incubation.

HEK293-TLR2 and HEK293-TLR4/MD2 co-transfectants were grown in RPMI-1640 supplemented with 10% fetal calf serum, 104 U/ml penicillin, 104 U/ml streptomycin, 2 mM l-glutamine, and 1 mg/ml G418 (Invitrogen) overnight at 37°C. For LPS or Pam3CSK4 content determination, a total of 10^5^ cells in 100 μl RPMI were plated onto 96-well flat-bottom plates and stimulated with a titration of LPS (20–1 ng/ml) or Pam3CSK4 (50–1 μg/ml). Subsequently, supernatants were analyzed for IL-8 production by ELISA.

### Naïve CD4 T Polarization Assay

Monocyte-derived DCs were stimulated with plate-bound FhTE in the presence or absence of LPS (10 ng/ml). After 48 h, expression of costimulatory molecules was measured by flow cytometry using the following antibodies: anti-CD86 (BU63), -CD83 (HB15e), -HLA-DR (L203), -CD40 (5C3), and -OX40L (Ik-1).

After stimulation, cells were washed and cocultured with allogenic naïve CD4 T cells (CD4^+^ CD45RA^+^, ratio 1:10), purified by MACS Beads (Miltenyi), in the presence of Staphylococcal Enterotoxin B (10 pg/ml, Sigma). On day 5, supernatants were harvested (for evaluation of IFNγ) and replaced with rhuIL-2 (100 U/ml, immunotools). Primed CD4^+^ T cells were stimulated with a cocktail containing 100 ng/ml Phorbol 12-myristate 13-acetate, 1 μg/ml ionomycin, and 10 μg/ml brefeldin A for 5–6 h. The cells were washed, fixed, and permeabilized using the Cytofix/Cytoperm kit (BD Biosciences) and subsequently stained with a combination of IL-4-PE and IFN-γ-FITC antibodies (BD Biosciences).

### CLR-Fc Binding Assay

NUNC maxisorp plates (Roskilde, Denmark) were coated with FhTE (125 μg/ml) overnight at 4°C. Plates were blocked with 1% bovine serum albumin (BSA) in TSM (20 mM Tris, pH 7.4, 150 mM NaCl, 1 mM CaCl_2_, and 2 mM MgCl_2_), and 1 μg/ml of different hCLR-Fc in TSM were added for 2 h at room temperature. Specific binding was blocked through the preincubation of hCLR-Fc with the Ca^2+^-chelator EGTA (10 mM). For hMGL-Fc, the specific binding was blocked with free GalNAc (100 mM; Sigma-Aldrich) or blocking anti-hMGL antibody (1G6.6, 10 μg/ml), by preincubation for 30 min at 37°C. Binding was detected using a peroxidase-labeled, anti-human IgG-Fc antibody (Jackson ImmunoResearch Laboratories, PA, USA). Binding was visualized with 3,3′,5,5′-tetramethylbenzidine (TMB) as a substrate (Sigma-Aldrich), and optical density was measured by spectrophotometry at 450 nm. When indicated, FhTE was pretreated with the enzymes α-*N*-acetylgalactosaminidase or α-manosidase (Prozyme, CA, USA), as indicated in manufacturer’s instructions.

### Western Blot

Proteins in FhTE were separated in a 15% SDS-PAGE and transferred to nitrocellulose sheets (Amersham, Saclay, France) at 45 V overnight in 20 mM Tris–HCl, pH 8.3, 192 mM glycine, and 10% ethanol. Residual protein-binding sites were blocked by incubation with 1% BSA in TSM at 37°C for 1 h. The nitrocellulose was then incubated for 1 h at room temperature with hMGL-, mMGL1-, or mMGL2-Fc in TSM. After three washes with TSM containing 0.1% Tween-20, the membrane was incubated for 1 h at room temperature with a peroxidase-labeled anti-human IgG-Fc antibody. For the oxidation of the glycan moieties of FhTE, strips were treated with 10 mM of sodium metaperiodate in 50 mM sodium acetate buffer pH 4.5 for 30 min at room temperature in the dark, washed with 50 mM sodium acetate buffer, and subsequently incubated for 1 h with glycine 1% at room temperature. As control, strips were subjected to the same treatment except for the incubation with sodium metaperiodate.

### Infections and Cell Cultures

Each BALB/c mouse was orally infected with 10 *F. hepatica* metacercariae (Baldwin Aquatics, USA). At 3 weeks postinfection (wpi), peritoneal exudates cells (PECs), spleens, and livers were removed. PECs were harvested by washing the peritoneal cavity with 5 ml of cold PBS. Purified CD11c^+^ cells from PECs of infected and non-infected animals were obtained by positive selection (StemCell Technologies, Canada). In all cases, a purity >90% was obtained. CD11c^+^ cells were stimulated with plate-bound FhTE, as indicated above. For mixed lymphocyte reactions, splenic CD4^+^ T cells were purified from C57BL/6 mice. For syngenic stimulation, purified splenic CD4^+^ T cells from BALB/c-infected animals sacrificed at 3 wpi were used. CD4^+^ T cells were cultured with stimulated CD11c^+^ cells for 5 days at 37°C and 5% CO_2_. IFN-γ and IL-10 levels were then quantified in the culture supernatants. Alternatively, cells were additionally stimulated with Pam2CysK4 (1 μg/ml) for 2 days at 37°C and 5% CO_2_.

Peritoneal exudate cells from infected and non-infected mice were washed twice with PBS containing 2% FBS and 0.1% sodium azide. The following antibodies were used in these experiments: anti-CD8 (53-6.7), -CD11c (N418), -I-A/I-E (2G9), -F4/80 (BM8), -CD86 (GL1), -CD11b (M1/70), -SIRPα (P84), -Ly6G (RB6-8C5), -Ly6C (HK1.4), -Siglec-F (E50-2440), -mMGL1 (LOM-8.7), and -mMGL2 (URA-1). Cells were then washed twice with PBS containing 2% FBS and 0.1% sodium azide and fixed with 1% formaldehyde. Cell populations were analyzed using a BD FACSCalibur (BD Biosciences). Antibodies were obtained from Affymetrix (CA, USA) or BD-Biosciences (CA, USA). IL-10 and IL-12/IL23p40 *in vivo* production and expression of CD68 (FA-11) were analyzed by intracellular staining. PECs from infected and non-infected mice were cultured for 6 h with GolgiPlug (BD Biosciences) when needed, washed, stained with CD11c, and then fixed and permeabilized using the Cytofix/Cytoperm kit (BD Biosciences) and subsequently stained with Abs specific for IL-12/23p40 or IL-10 (Biolegend, CA, USA).

### Internalization Assay

The internalization and binding of FhTE to CD11c^+^ cells in PECs were analyzed by flow cytometry. Briefly, PECs (1 × 10^5^/well) were incubated with Alexa 647-labeled FhTE for 1 h at 37°C in complete medium (to assess uptake), or at 4°C in complete medium (to assess binding). Cells were then washed twice and the binding or internalization by CD11c^+^ cells was analyzed by FACS.

### Quantitative Real-time RT-PCR

Total RNA was isolated from spleen, liver, and purified CD11c^+^ cells from PEC by using Tri-reagent (Sigma-Aldrich). Quantitative real-time PCR was performed in StepOne™ real-time PCR system (Applied Biosystems) using Fast SYBR^®^ Green Master Mix (Applied Biosystems) (25). The reactions were performed according to the following settings: 95°C for 20 s min for initial activation, followed by 40 thermal cycles of 3 s at 95°C, and 30 s at 60°C. All reactions were performed with five biological and two technical replicates with negative controls.

### Microscopy Analyses

Livers from infected mice after 3 wpi or naive mice (control) were harvested, embedded in OCT, and snap frozen in nitrogen. Sections were cut at a thickness of 8 μm, fixed with cold acetone for 10 min, and blocked with 5% BSA in 3% rat serum for 1 h at room temperature. Sections were then overnight incubated at 4°C with anti-mMGL2 (URA-1), -cCD11c (N418), and -F4/80 (BM8), stained with 4′,6-diamidino-2-phenylindole and visualized in an epifluorencense microscope Olympus IX-81 and confocal microscope Leica TCS-SP5-II.

### Statistical Analysis

Results were analyzed using a one-way ANOVA followed by Bonferroni Multiple Comparison test or a student’s *t*-test using GraphPad Prism software (GraphPad Software, San Diego, CA, USA). Results were considered to be significantly different when *p* < 0.05.

## Results

### *F. hepatica* Glycoconjugates Potentiate the TLR2- and TLR4-Induced Production of TNFα and IL-10 by mo-DCs *via* hMGL

**Figure 1:**
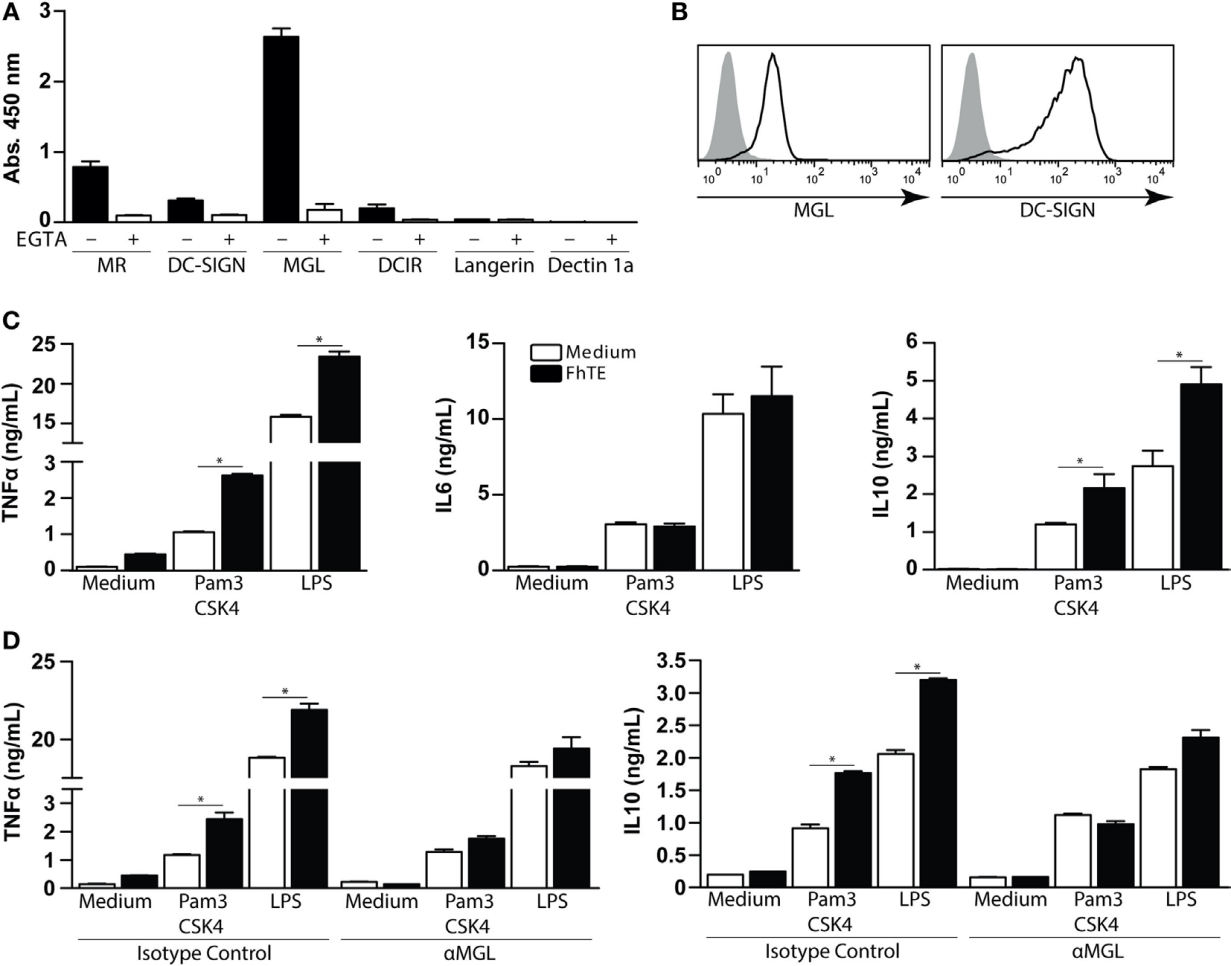
**Macrophage Gal/GalNAc lectin recognizes *F. hepatica* glycoconjugates and potentiates the production of IL-10 and TNF-α by Pam3CSK4-stimulated monocyte-derived DCs (mo-DCs)**. **(A)** Binding of different hCLR-Fc on FhTE-coated plates in presence (white bars) or absence (black bars) of EGTA. **(B)** Expression of hMGL and DC-SIGN (as differentiation marker) on mo-DCs was confirmed by flow cytometry. **(C)** IL-6, IL-10 and TNFα levels on supernatants from Pam3CSK4 and LPS-stimulated mo-DC cultures incubated with and without FhTE. **(D)** moDCs were stimulated as in **(B)**, in the presence of an anti-hMGL antibody or isotype control. A representative result of one out of four donors is shown (±SEM, indicated by error bars). Asterisks indicate statistically significant differences (*p < 0.05).

Several studies have demonstrated that different parasites modulate the host immune response through the interaction with CLRs expressed on immune cells (16, 18, 26). In order to evaluate the involvement of different CLRs in the recognition of *F. hepatica* glycoconjugates, we performed an ELISA-like assay coating *F. hepatica* components on the plate and further incubating them with a variety of CLRs-Fc fusion proteins. FhTE was highly recognized by hMGL and, to a lower extent, by Mannose receptor (MR), DC-SIGN, and DCIR, while it was not recognized by Dectin-1 or Langerin (Figure 1A). As expected, the observed CLR binding was abrogated in presence of the chelating agent EGTA, indicating that divalent cations such as Ca^2+^ are essential for this interaction/binding.

Given that hMGL strongly interacted with FhTE and that hMGL triggering modulates the TLR-induced maturation of mo-DCs (27), we sought to evaluate whether FhTE was able to modulate DC maturation *via* this CLR. To this end, we first confirmed the expression of hMGL on mo-DCs (Figure 1B). mo-DCs were then cultured on FhTE-coated plates in the presence or absence of Pam3CSK4 (TLR1/2 ligand) or LPS (a TLR4 ligand), and the production of different cytokines was evaluated in the culture supernatants. Although FhTE did not induce the expression of TNFα, IL-6, and IL-10, it enhanced the production of TNFα and IL-10, but not IL-6, by Pam3CSK4- and LPS-stimulated mo-DCs (Figure 1C). Interestingly, an anti-hMGL blocking antibody abrogated the enhanced production of TNFα and IL-10, indicating that there is a crosstalk between TLR1/2/4 and hMGL in the presence of parasite components (Figure 1D). Of note, the hMGL-mediated crosstalk was only detected when mo-DCs were cultured with immobilized, but not soluble, FhTE (Figure S2 in Supplementary Material), suggesting that hMGL cross-linking is required for triggering or that internalization is dispensable for hMGL triggering, as already reported for other hMGL ligands (28).

### Triggering of MGL by *F. hepatica* Antigens Induce Th2 Polarization by Reduction of IFNγ

**Figure 2:**
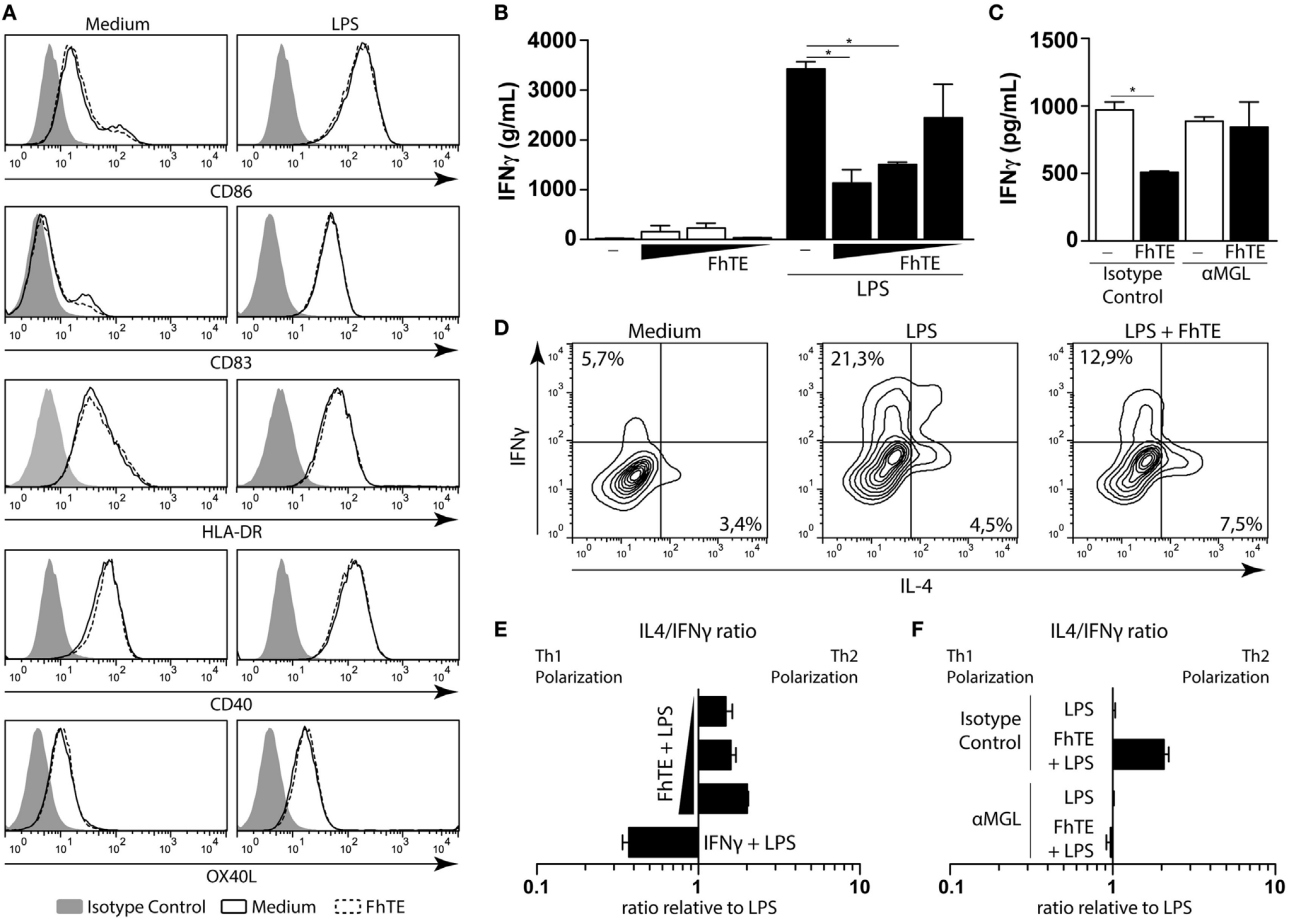
**FhTE induces Th2 polarization by reducing IFNγ and increasing IL-4 production**. moDCs were incubated with coated FhTE in the presence or absence of LPS (10 μg/ml) for 48 h and the expression of costimulatory molecules was evaluated by flow cytometry **(A)**. To evaluate the capacity of the stimulated moDCs to polarize naïve CD4 T cells, cells were washed and incubated with CD4^+^ CD45RA^+^ T cells (ratio 1:10) in the presence of Staphylococcal Enterotoxin B (10 pg/ml). After 5 days, supernatants were harvested for the evaluation of IFNγ **(B,C)**, and replaced with 100 U of IL-2. After 5 days, Th1/Th2 polarization of T cells was evaluated by intracellular staining of IFNγ and IL-4 after stimulation with PMA and Ionomycin in the presence of Brefeldin A **(D–F)**. When indicated **(C,F)**, moDCs were preincubated with an anti-hMGL antibody or an isotype control, before stimulation. IL-4/IFNγ ratio was evaluated relative to the control, based in single positive cells. Concentration of FhTE used: 200, 100, and 50 μg/ml. A representative result of one out of four donors is shown (±SEM, indicated by error bars). Asterisks indicate statistically significant differences (*p < 0.05).

Next, we evaluated whether MGL triggering by *F. hepatica* on DCs could modulate the differentiation of T cells. Thus, we analyzed the costimulatory markers on LPS-matured mo-DCs conditioned with *F. hepatica* components. Interestingly, FhTE was unable to induce any change in the expression of costimulatory markers on LPS-matured mo-DCs (Figure 2A). However, these DCs induced lower production of IFNγ by stimulated T cells in a dose-dependent manner as compared to control LPS-matured mo-DCs (Figure 2B). In addition, the reduced capacity to induce IFNγ-producing T cells by FhTE/LPS-matured mo-DCs was abrogated by the anti-MGL antibody (Figure 2C). Finally, mo-DCs matured in the presence of LPS and FhTE polarized T cells toward a Th2 phenotype, since they produced higher IL-4/IFNγ ratio than LPS-stimulated mo-DCs (Figures 2D–E). Importantly, the capacity of FhTE/LPS-stimulated mo-DCs to induce Th2 polarization was mediated by MGL since a specific anti-MGL antibody abrogated this process (Figure 2F).

### hMGL Interacts with the Tn Antigen Present on FhTE

**Figure 3:**
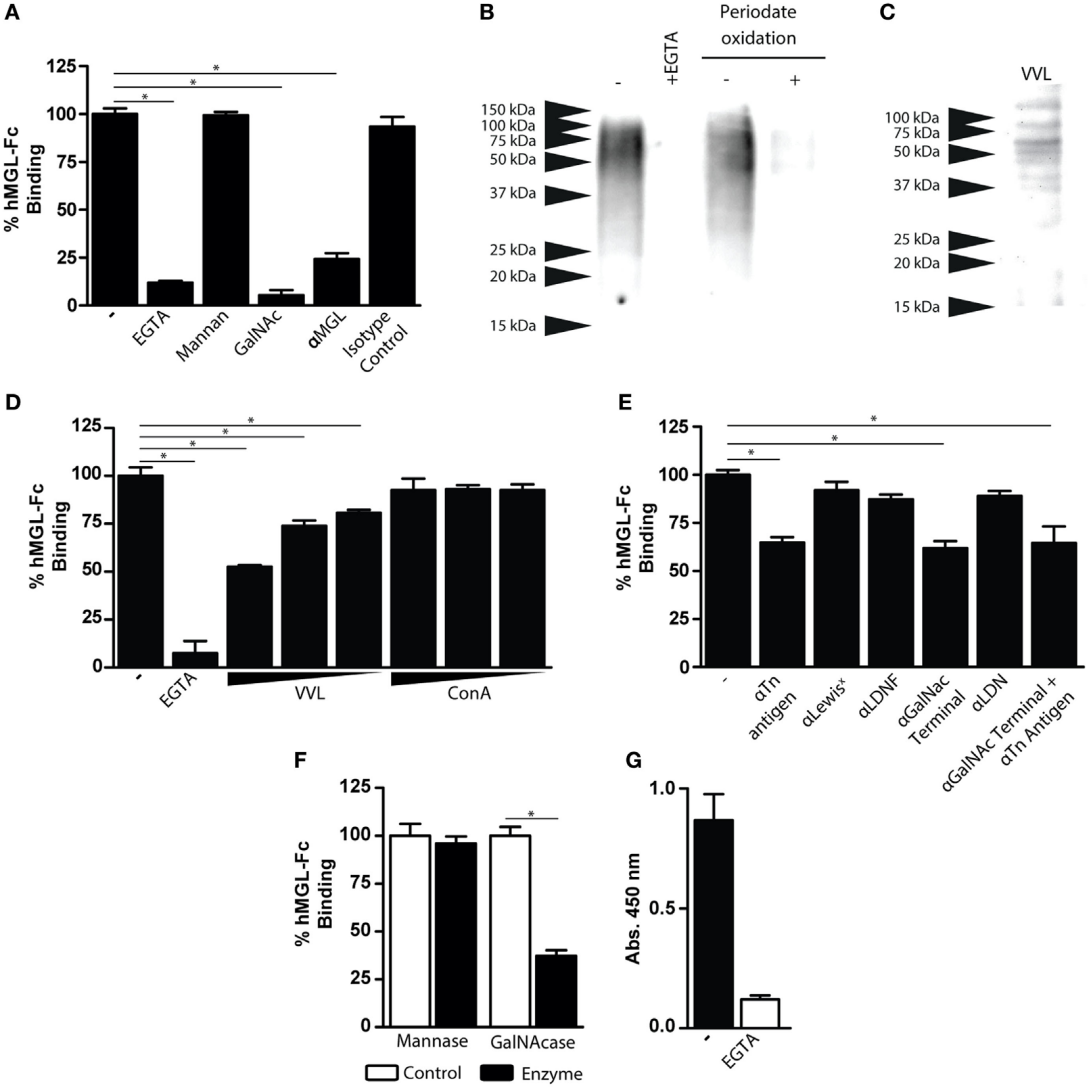
**Characterization of FhTE glycoconjugates that are recognized by hMGL**. **(A)** hMGL binding was evaluated on FhTE-coated plates with hMGL-Fc previously incubated with EGTA, mannan, GalNAc, anti-hMGL antibody, or isotype control. **(B)** Western Blot with hMGL-Fc on FhTE. Recognition of FhTE by hMGL was abrogated with EGTA and with periodate oxidation treatment of FhTE. **(C)** Lectin blot of FhTE using the GalNAc-specific lectin *Vicia Villosa* (VVL). **(D)** Inhibition of hMGL binding to plate-bound FhTE with the lectins VVL and ConA (1, 0.1, and 0.1 μg/ml). **(E)** Inhibition of hMGL binding to plate-bound FhTE with different carbohydrate-specific antibodies. **(F)** hMGL binding on FhTE-coated plates previously incubated with mannase or GalNAcase. **(G)** Binding of hMGL on membrane-associated parasite component-coated plates in presence (white bars) or absence (black bars) of EGTA. A representative figure of three or four independent experiments is shown (±SD, indicated by error bars). Asterisks indicate statistically significant differences (*p < 0.05).

To identify the nature of *F. hepatica* glycoconjugates recognized by hMGL, we carried out binding inhibition assays and selective deglycosylation to abolish hMGL recognition of FhTE. hMGL binding was abrogated in the presence of EGTA, GalNAc, and an anti-hMGL blocking antibody, while it was not modified by incubation with mannan or the isotype control (Figure 3A). *F. hepatica* glycoconjugates recognized by hMGL were identified by western blotting using hMGL-Fc as a group of components ranging from 50 to 100 kDa (Figure 3B). Interestingly, hMGL-Fc reactivity was inhibited in the presence of EGTA and after metaperiodate oxidation of FhTE glycans (Figure 3B), confirming that the recognition of hMGL of FhTE glycoconjugates was glycan mediated. To confirm that GalNAc residues are present in FhTE, we carried out a lectin blot using the GalNAc-specific lectin from *Vicia Villosa* (VVL). VVL recognized molecular components with an apparent molecular weight pattern similar to that observed for hMGL (Figures 3B,C). Moreover, preincubation with the lectin VVL, but not ConA, two lectins that strongly interact with FhTE (10), inhibited the hMGL recognition, suggesting that VVL and hMGL interact with the same ligands present in FhTE (Figure 3D).

In order to establish the nature of the GalNAc-containing glycans present on FhTE that are recognized by hMGL, specific antibodies against the Tn antigen (αGalNAc-Thr/Ser), LDNF [GalNAcβ1-4(Fucα1-3)GlcNAc-R], LDN (GalNAcβ1-4GlcNAc-R) and Lewis^x^ [Galβ1-4(Fucα1-3)GlcNAc-R] were used. As shown in Figure 3E, only the anti-Tn and anti-GalNAc antibodies were able to reduce MGL binding to FhTE, while the blocking antibodies specific for the Lewis^x^, LDNF and LDN structures did not inhibit hMGL binding to FhTE (Figure 3E). In addition, hMGL binding to FhTE was inhibited after GalNAcase, but not mannase treatment of FhTE, indicating that hMGL recognizes terminal GalNAc residues present on FhTE (Figure 3F). Altogether, these results suggest that hMGL recognizes the Tn antigen present in FhTE.

The fact that MGL triggering induced by FhTE was only observed when FhTE was immobilized on plates suggests that MGL could recognize immobilized ligands present on the surface of the parasite. Thus, we investigated whether hMGL ligands are present in a tegumental extract of *F. hepatica*. Indeed, hMGL recognized FhTeg in a Ca^2+^-dependent manner (Figure 3G).

### mMGL2^+^ CD11c^+^ Cells Are Recruited to the Peritoneum and Liver of *F. hepatica*-Infected Mice

**Figure 4:**
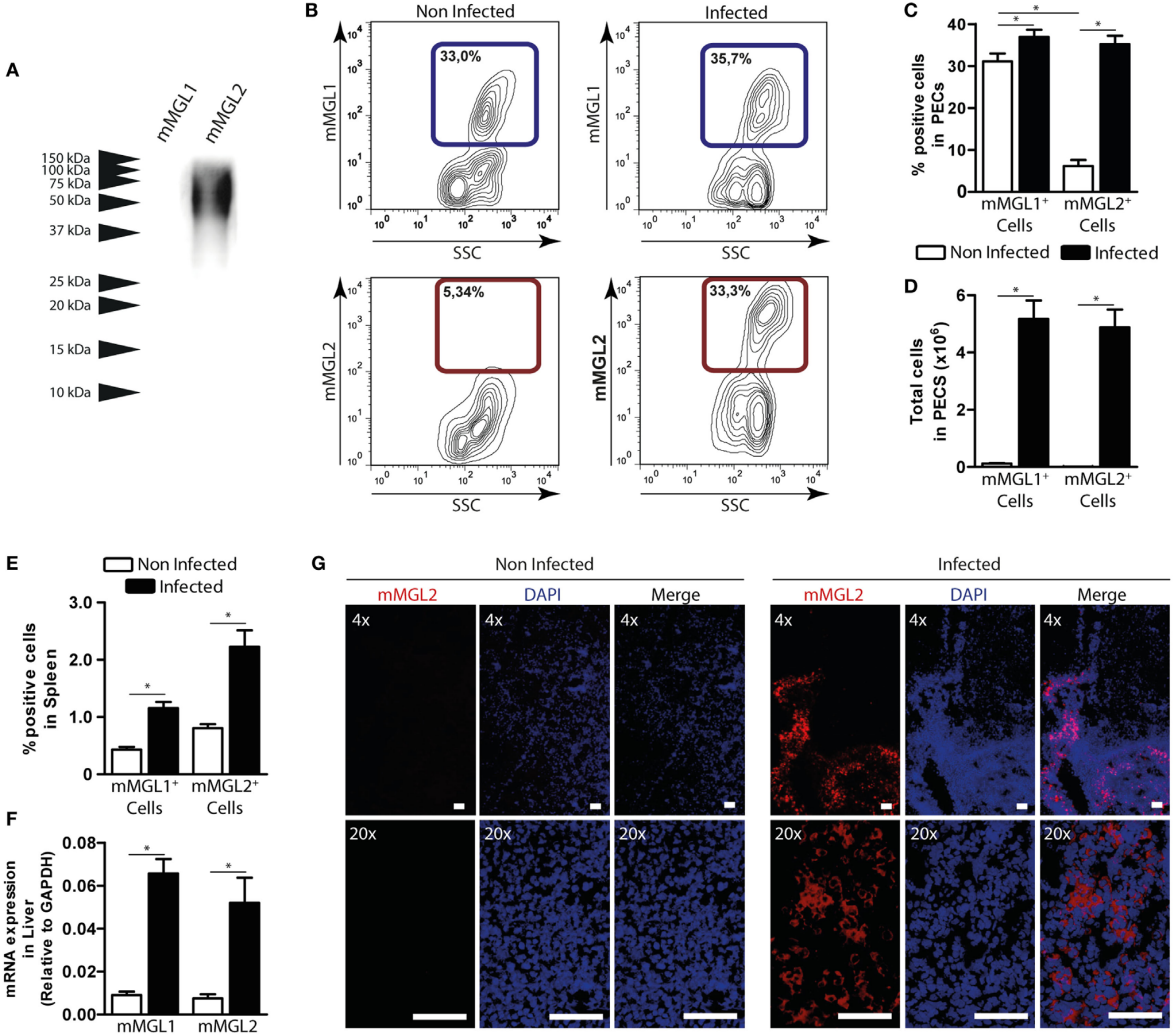
**mMGL2^+^ cells are recruited to the peritoneum during *F. hepatica* infection**. **(A)** Recognition of mMGL1 and mMGL2 of FhTE by western blotting. **(B)** Evaluation by flow cytometry of mMGL1^+^ and mMGL2^+^ cells in the peritoneal cavity of infected and control mice. **(C)** Percentage of mMGL1^+^ or mMGL2^+^ cells in the peritoneum of infected and control mice. **(D)** Total cell numbers of mMGL1^+^ or mMGL2^+^ cells in the peritoneum of infected and control mice. **(E)** Percentage of mMGL1^+^ or mMGL2^+^ cells in the spleen of infected and control mice. **(F)** mRNA expression of mMGL1 and mMGL2 in the liver of infected and control animals. **(G)** MGL2 expression in livers of infected and control animals. A representative figure of three independent experiments is shown (±SD, indicated by error bars). Asterisks indicate statistically significant differences (*p < 0.05). The bar represents 100 μm.

In order to get more insights into the recognition of *F. hepatica* glycoconjugates by MGL*^+^* cells during parasite infection, we orally infected mice with *F. hepatica* metacercarie and analyzed the expression of mMGL1 and mMGL2 on cells from spleen, PECs, and liver after 3 wpi. Since mice possess two isoforms of the MGL receptor: mMGL1 and mMGL2 (15), we first evaluated whether they can recognize glycoconjugates present on FhTE by Western blotting. As shown in Figure 4A, only mMGL2 recognized parasite components ranging from 37 to 100 kDa in migratory pattern similar to the one observed for hMGL, while mMGL1 did not recognize any *F. hepatica* components.

Interestingly, mMGL2*^+^* cells significantly increased in infected mice, while no changes were observed in the percentage of mMGL1*^+^* PECs (Figures 4B,C and Figure S3A in Supplementary Material). In contrast, when analyzing the total amount of cells, both mMGL1*^+^* and mMGL2*^+^* PECs increased in infected animals (Figure 4D), probably due to the great recruitment of cells in the peritoneum upon infection. On the other hand, both mMGL1*^+^* and mMGL2*^+^* cells were augmented in spleen (Figure 4E). Moreover, mMGL1 and mMGL2 expression evaluated by qRT-PCR in livers from infected and non-infected mice showed that both isoforms were overexpressed in this tissue during infection (Figure 4F). Finally, the recruitment of mMGL2*^+^* cells was evaluated by microscopy on liver sections, indicating the presence of these cells in the leukocyte infiltrate of infected animals, but not control mice (Figure 4G and Figure S3B in Supplementary Material).

**Figure 5:**
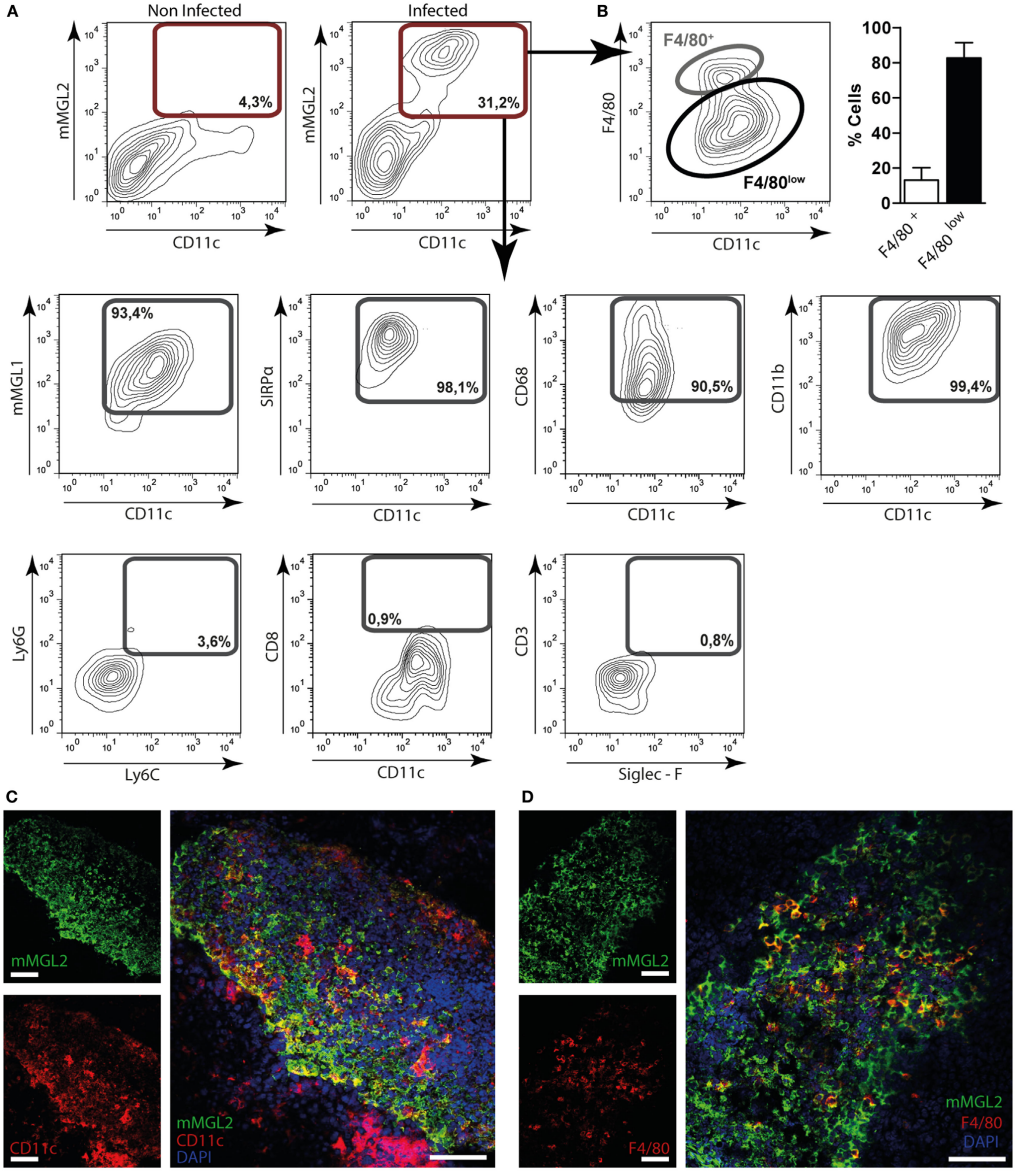
**Immunephenotyping of mMGL2^+^ cells in the peritoneum and in the liver of *F. hepatica*-infected animals**. **(A)** Cells from the peritoneal cavity from infected or control mice were stained with CD11c-, mMGL1-, mMGL2-, CD11b-, CD8-, F4/80-, SIRPα-, CD68-, Ly6G-, Ly6C-, CD3-, and Siglec-F-specific antibodies and evaluated by flow cytometry. **(B)** Percentage of mMGL2^+^ CD11c^+^ F4/80^+^ or mMGL2^+^ CD11c^+^ F4/80^low^ cells from PECs of infected animals. **(C)** Expression of MGL2^+^ and CD11c^+^ cells in the liver of infected mice. **(D)** Expression of MGL2^+^, CD11c^+^ and F4/80^+^ cells in the liver of infected mice. The bar represents 100 μm. A representative figure of three independent experiments is shown (±SD, indicated by error bars).

In order to characterize the mMGL2*^+^* cell population present in the peritoneum of infected mice, we performed phenotype analyses by flow cytometry. As shown in Figure 5A, CD11c*^+^* cells from infected mice, but not control mice, expressed mMGL2. These cells also expressed mMGL1, CD11b, SIRPα, and CD68, while they did not express CD8, Ly6G, Ly6C, CD3, or Siglec-F (Figure 5A). Furthermore, we observed that they were mainly characterized by a low expression of F4/80 (Figure 5B). Last, confocal microscopy of liver sections indicated that some of the mMGL2*^+^* cells present in the leukocyte infiltrate of infected livers expressed CD11c (Figure 5C) and F4/80 (Figure 5D).

### mMGL2^+^ CD11c^+^ Cells from *F. hepatica*-Infected Animals Expand IL-10-Producing CD4^+^ T Cells and Suppress Th1 Immune Responses

**Figure 6:**
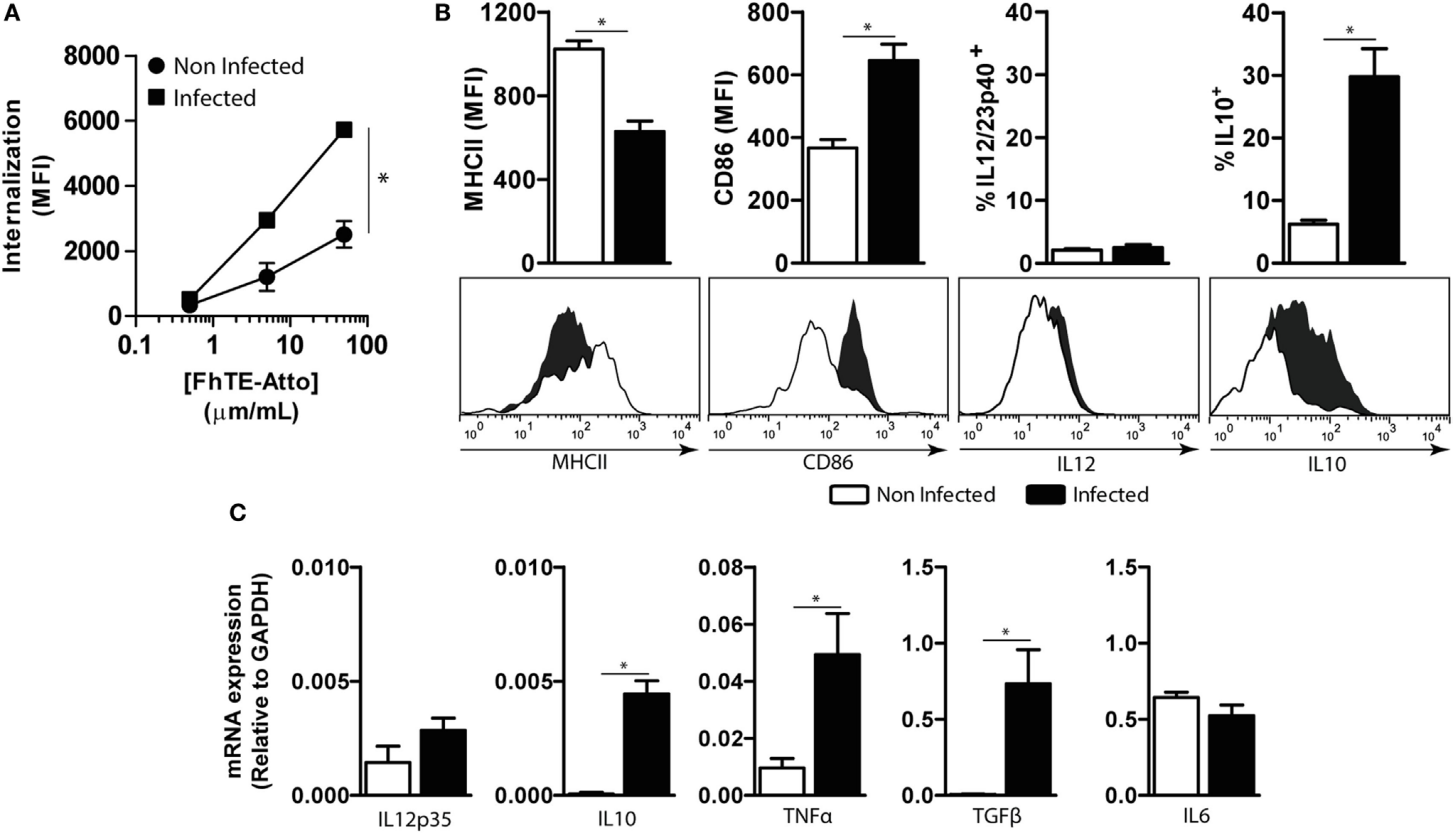
**Characterization of CD11c^+^ mMGL2^+^ cells**. CD11c^+^ cells were purified from the peritoneal cavity of infected mice at 3 wpi and naive mice. **(A)** Uptake was evaluated by flow cytometry on PECs previously incubated with different concentrations of Atto 647-labeled FhTE for 1 h at 37°C or 4°C as a control. Internalization is calculated as the difference between the MFI at 37°C and MFI at 4°C. **(B)** The expression of MHCII and CD86 and the production of IL-12/23p40 and IL-10 by CD11c^+^ purified cells were evaluated by flow cytometry. **(C)** Expression of IL-6, IL-12p35, IL-10, TNFα, and TGFβ was evaluated by qRT-PCR on RNA obtained from purified CD11c^+^ cells. A representative figure of three independent experiments is shown (±SD, indicated by error bars). Asterisks indicate statistically significant differences (*p < 0.05).

In order to establish whether mMGL2^+^ CD11c^+^ cells are immunomodulated by *F. hepatica*, we first evaluated their capacity to take up parasite components and secrete cytokines. To this end, PECs from infected and non-infected animals were incubated with Atto647-labeled FhTE and evaluated by flow cytometry in CD11c^+^ cells (Figure 6A). Peritoneal mMGL2^+^ CD11c^+^ cells from infected animals presented a higher capacity of FhTE internalization than CD11c^+^ cells from non-infected animals. In addition, they expressed MHC II and CD86; while MHC II was expressed at lower levels than CD11c*^+^* cells from non-infected mice, CD86 was upregulated in MGL2^+^ cells from infected mice (Figure 6B). Furthermore, they produced higher transcript levels of the regulatory cytokines IL-10, TNFα, and TGFβ, while no differences were observed in the transcript levels of IL-6, IL-12/23p40, or IL-12p35 (Figures 6B,C), suggesting a potential regulatory role of these cells during infection.

**Figure 7:**
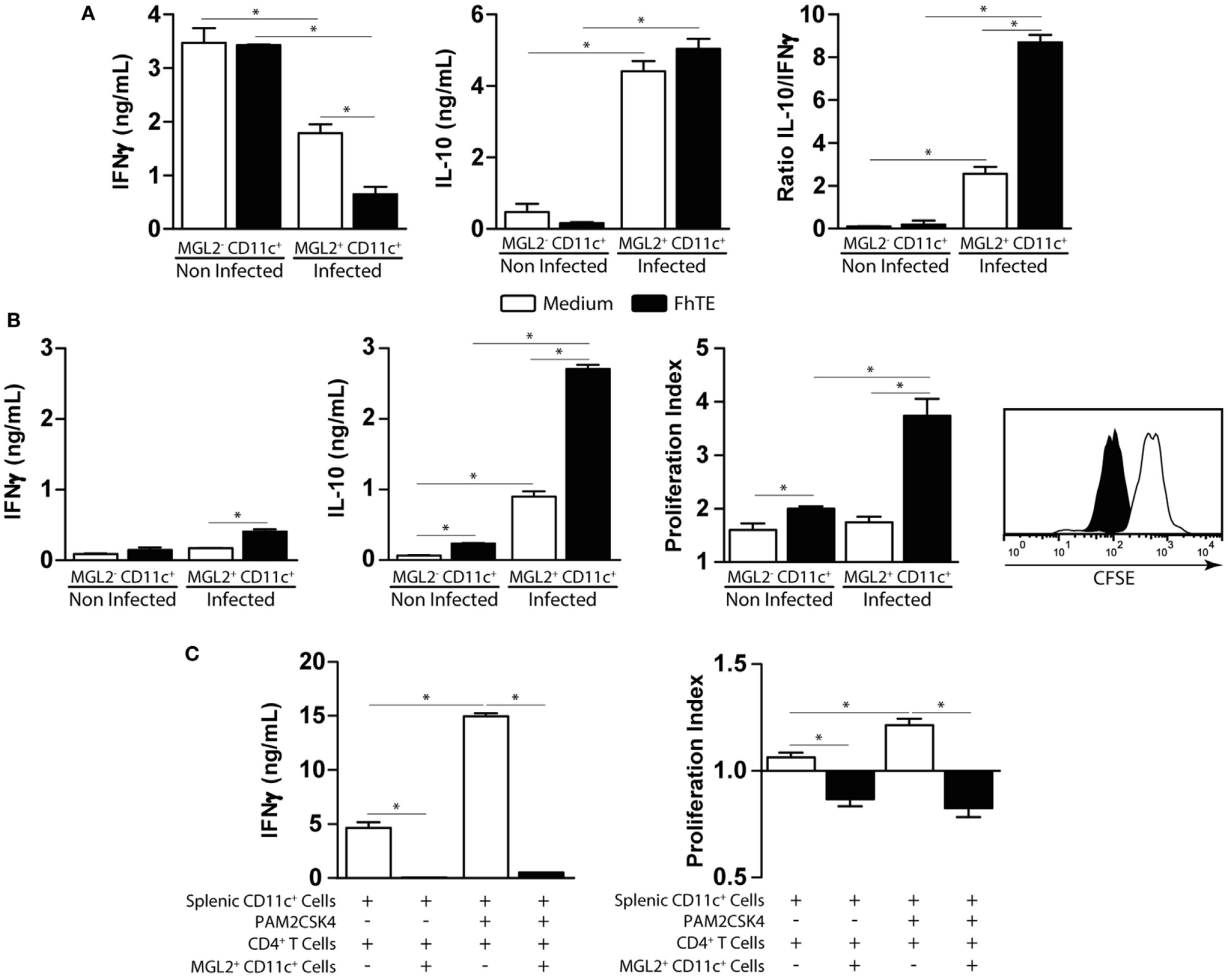
**Functional analyses of CD11c^+^ mMGL2^+^ cells**. **(A)** CD11c^+^ purified cells from the peritoneal cavity of infected or control mice were stimulated with FhTE-coated plates overnight at 37°C, washed and incubated with purified C57BL/6 CD4^+^ T cells from naive animals for 5 days at 37°C. IFNγ and IL-10 production by T cells was evaluated on culture supernatants by ELISA. **(B)** CD11c^+^ purified cells from the peritoneal cavity of infected or control mice were stimulated with FhTE overnight at 37°C, washed and incubated with purified BALB/c CD4^+^ T cells from 3-week-infected animals for 5 days at 37°C. IFNγ and IL-10 production by T cells was evaluated on culture supernatants by ELISA. Proliferation was evaluated on CFSE-stained CD4^+^ T cells by flow cytometry. The proliferation index was calculated as the ratio between the percentage of CFSE^low^ CD4^+^ cells and CFSE^low^ CD4^+^ cells with medium. **(C)** CD11c^+^ purified cells from the spleen of naïve BALB/c mice were stimulated with Pam2CSK4 in the presence or absence of FhTE overnight at 37°C, washed and incubated with purified C57BL/6 CD4^+^ T cells from naive animals for 5 days at 37°C in presence or absence of MGL2^+^ CD11c^+^ cells (100,000/well) from 3-week-infected animals. IFNγ production by T cells was evaluated on culture supernatants by ELISA. Proliferation was evaluated on CFSE-stained CD4^+^ T cells by flow cytometry. The proliferation index was calculated as the ratio between the percentage of CFSE^low^ CD4^+^ cells and CFSE^low^ CD4^+^ cells with medium. A representative figure of three independent experiments is shown (±SD, indicated by error bars). Asterisks indicate statistically significant differences (*p < 0.05).

Then, we evaluated the T-cell stimulatory capacity of mMGL2^+^ CD11c^+^ cells both in allogenic and syngenic cultures using purified CD4^+^ T cells. mMGL2^+^ CD11c^+^ cells from infected animals induced the production of IL-10 and a decrease of IFNγ secretion by allogenic CD4^+^ T cells as compared with mMGL2^−^ CD11c^+^ cells from non-infected animals (Figure 7A). Interestingly, mMGL2^+^ CD11c^+^ cells (but not naive mMGL2^−^ CD11c^+^) enhanced the IL-10/IFNγ production ratio by CD4^+^ T cells (Figure 7A). Of note, the IL-10/IFNγ production ratio by CD4^+^ T cells was significantly increased when mMGL2^+^ CD11c^+^ cells were previously stimulated with coated FhTE (Figure 7A). mMGL2^+^ CD11c^+^ cells from infected animals also expanded specific CD4^+^ T cells that produced high levels of IL-10 in the absence of IFNγ (Figure 7B) and their function was enhanced when these cells were used with FhTE (Figure 7B), indicating that mMGL2^+^ CD11c^+^ cells induce IL-10^hi^ IFNγ^low^ CD4^+^ T cells, in both antigen-dependent and -independent manner. Last, we evaluated the capacity of mMGL2^+^ CD11c^+^ cells to suppress the induction of Th1 immune responses. To this end, purified splenic CD11c^+^ cells from naive mice were stimulated with PAM2CSK4 and incubated with allogenic CD4^+^ T cells in the presence or absence of mMGL2^+^ CD11c^+^ cells from infected mice. As shown in Figure 7C, mMGL2^+^ CD11c^+^ cells inhibited both the proliferation and the production of IFNγ by CD4^+^ T cells. Altogether, these results show that mMGL2^+^ CD11c^+^ cells from infected mice expand IL-10 producing CD4^+^ T cells and suppress Th1 differentiation.

**Figure 8:**
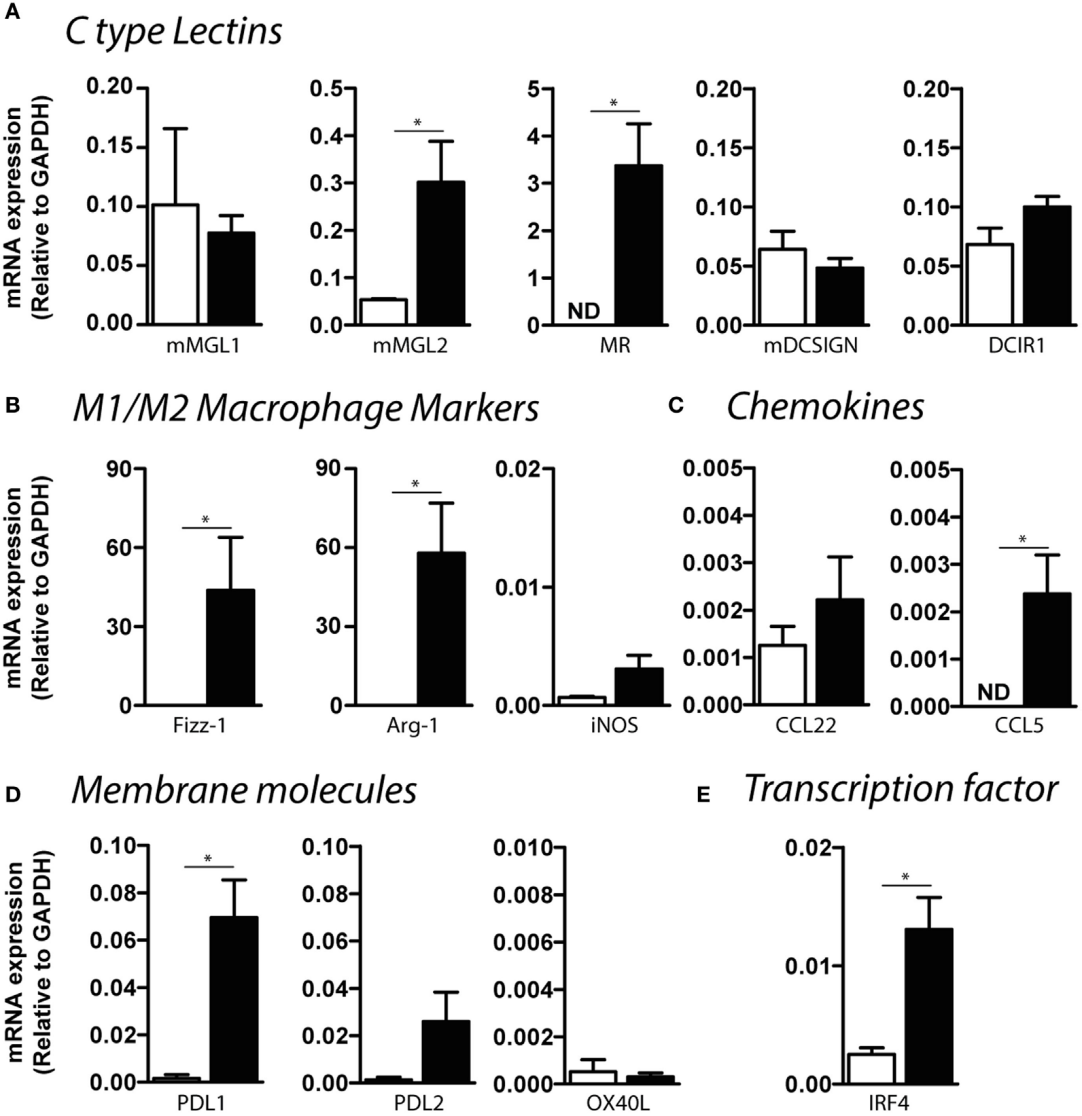
**Expression of C-type lectin receptors, chemokines, and regulatory molecules by CD11c^+^ mMGL2^+^ cells**. Expression by qRT-PCR of CLRs **(A)**, macrophage markers **(B)**, chemokines **(C)**, regulatory membrane molecules **(D)**, and IRF-4 **(E)** was evaluated on purified peritoneal CD11c^+^ cells from infected (black bars) and control (white bars) mice. A representative figure of three independent experiments is shown (±SD, indicated by error bars). Asterisks indicate statistically significant differences (*p < 0.05).

In order to deeply understand the immunoregulatory function of mMGL2^+^ CD11c^+^ cells from infected mice, we evaluated the expression of a variety of molecules that might participate in different DC functions and compare them with mMGL2^−^ CD11c^+^ cells purified from naive control mice. CD11c^+^ cells from infected mice expressed high levels of mMGL2 and the MR than CD11c^+^ cells from control mice (Figure 8A). Moreover, these cells also expressed increased levels of Fizz-1 and Arg-1 (Figure 8B), commonly associated with alternative activated macrophages. However, no differences in the expression of inducible nitric oxide synthase, associated with a pro-inflammatory activation of macrophages was observed between CD11c^+^ cells from infected and control mice (Figure 8B). CD11c^+^ cells from infected mice also overexpressed CCL5 (Figure 8C), as well as other immunomodulatory molecules like the PD-L1 (Figure 8D) and the transcription factor interferon regulatory factor 4 (IRF4, Figure 8E), known to control Th2 cell differentiation. Altogether, our results suggest that MGL2^+^ CD11c^+^ cells are recruited to the peritoneal cavity during *F. hepatica* infection, acquiring different regulatory markers associated to regulatory macrophages and potentially regulating the T cell polarization to a Th2/regulatory phenotype.

## Discussion

In this work, we provide evidence that *F. hepatica* immune modulates CD11c^+^ MGL^+^ cells during infection and that they contribute to the expansion of IL-10-producing T cells and the suppression of Th1-polarized immune responses. Parasite components also modulated the TLR-induced maturation of DCs through the binding of Tn carbohydrate structures to the C-type lectin MGL, indicating that this receptor is a key player in the immunoregulatory mechanisms triggered by *F. hepatica*. The capacity of MGL to act as a pattern recognition receptor has already been described. Indeed, MGL can recognize glycosylated molecules expressed by bacteria such as *Neisseria gonorrhoeae* (29), *Bordetella pertussis* (30), and *Campylobacter jejuni* (31, 32), as well as virus, including the Ebola (33) and Influenza (34). Furthermore, helminth-derived molecules can also interact with MGL. For instance, MGL recognizes *S. mansoni* through LacdiNAc residues (20, 35), while it binds *T. suis* through terminal αGalNAc residues (21). These evidences, together with our results, highlight the role of MGL in mediating pathogen-triggered immunoregulatory strategies.

We here show that the interaction with *F. hepatica*-derived molecules triggers MGL signaling that, together with TLR-triggering, results in an enhanced production of IL-10 and TNFα. While the MGL-induced production of IL-10 and TNFα by human MGL^+^ DCs has already been described (28, 36), this is the first report demonstrating that the Tn antigen present in *F. hepatica* components interacts with MGL on DCs and favors Th2 polarization. It is well known that IL-10 is a cytokine with potent anti-inflammatory properties that plays a central role in limiting host immune response to pathogens. Also, TNFα, although classically grouped as a pro-inflammatory cytokine, can have inhibitory properties, especially when associated with IL-10. Indeed, tolerogenic DCs generated in the presence of vitamin D3, secrete high amounts of both IL-10 and TNFα after LPS activation and favor the expansion of regulatory T cells in a TNFα-dependent manner (37). MGL^+^ DCs can also instruct the differentiation of T cells toward Tr1 regulatory cells in an IL-10-dependent manner (36). This phenomenon could constitute a mechanism used by *F. hepatica* to evade immunity, as has already been proposed for *C. jejuni*, where MGL inhibits the upregulation of DC-maturation marker expression and limits production of the pro-inflammatory cytokine IL-6 (31).

However, most of the published studies describe the immunomodulatory role of MGL in *in vitro* settings. In order to evaluate whether MGL plays a role in DC immunomodulation during infection with *F. hepatica*, we analyzed the phenotypic and functional characteristics of MGL^+^ DC in *F. hepatica*-infected mice. Although both mMGL1^+^ and mMGL2^+^ cells increased in the peritoneal cavity, spleen, and liver of infected mice, the proportion of mMGL2^+^ cells were clearly augmented both in PEC, spleen, and liver, suggesting either a recruitment of these cells or a strong increase of mMGL2 expression induced during the infection. Of note, the tolerogenic stimulus dexamethasone induced the expression of hMGL on mo-DCs during DC-differentiation (24), suggesting a role in the development or maintenance of tolerance. In this scenario, we could speculate that mMGL2, but not mMGL1, seems to have a role during the infection process, since only mMGL2 was able to recognize parasite components.

Upon *F. hepatica* infection, we detected a recruitment of mMGL2^+^ cells in the peritoneum. These cells also expressed mMGL1, CD11c, CD11b, SIRPα, and CD68. However, they expressed low levels of F4/80, often used as macrophage marker (38), but also expressed by inflammatory DCs and CD11b lineage DCs (39). Similarly, although CD68 is highly expressed by monocytes and tissue macrophages, it can also be present to a lesser extent on DCs and peripheral blood granulocytes (40). Since CD68 has been implicated in the mediation, recruitment, and activation of macrophages (41, 42), it would be interesting to determine whether it can participate in the recruitment of mMGL2^+^ cells to the peritoneum or liver of infected mice. Furthermore, the phenotype found for mMGL2^+^ cells in *F. hepatica*-infected mice suggests that they might correspond to DCs since the same phenotype was described in mMGL1^+^ mMGL2^+^ cells in lung, spleen, and bone marrow from naïve mice, while mMGL1 is expressed by a heterogeneous group of cells including, macrophages, cDC, and pDC (19). Experiments evaluating the expression of macrophage-specific molecules in mMGL2^+^ cells during *F. hepatica* infection, such as CD64 and MerTK (40), will determine whether these cells are macrophages or DCs.

mMGL2^+^ CD11c^+^ cells also expressed signal regulatory protein α (SIRPα), a regulatory membrane glycoprotein abundant in DCs, macrophages, and neutrophils that participates in immune homeostasis (43). It has been proposed that SIRPα^+^ DCs can regulate immune responses through its cytoplasmic region containing immunoreceptor tyrosine-based inhibition motifs. Indeed, SIRPα^+^ DCs can promote Th2-mediated allergic inflammation (44) and participate in the development of central tolerance against circulating peripheral antigens (45). In addition, ligation of SIRPα to its ligand CD47 suppresses DC maturation and inhibits cytokine production by mature DCs (46), suggesting that SIRPα can prevent activation of DCs. Peritoneal mMGL2^+^ CD11c^+^ cells were also characterized by decreased levels of MHCII, but increased expression of CD86, corresponding to the semi-mature phenotype of DCs already described for *F. hepatica* (10).

During *F. hepatica* infection, the mMGL2^+^ CD11c^+^ cells seem to acquire a regulatory program that activates specific IL-10-producing CD4^+^ T cells that correlates with the T cell response already described in animals infected with this parasite (4, 5, 10). Indeed, mMGL2^+^ CD11c^+^ cells, but not mMGL2^−^ CD11c^+^ cells from non-infected mice, produced the inmmunoregulatory cytokines TGFβ, IL-10, and TNFα that were associated with their capacity to activate IL-10-producing both allogenic and syngenic CD4^+^ T cells. Finally, FhTE-loaded mMGL2^+^ CD11c^+^ cells induced higher production of IL-10 by both allogenic and syngenic CD4^+^ T cells, suggesting that parasite components enhance the immunoregulatory program on DCs that regulate DC maturation and stimulatory function. Several reports are in agreement with our results, having already described that murine MGL2^+^ DCs are required for efficient Th2 development of mice infected with the hookworm *Nippostrongylus brasiliensis* (47). In addition, human MGL^+^ DCs exposed to *N. gonorrhoeae* LPS carrying a terminal GalNAc residue are prone to induce Th2-type T cells (29). Taken together, these and our observations suggest that MGL^+^ DCs induce and/or expand Th2 immune responses by triggering MGL.

The capacity of mMGL2^+^ CD11c^+^ cells to induce IL-10-producing T cells was associated to the expression of FIZZ1 and IRF4 by these cells. FIZZ1 is induced during Th2 cytokine immune response upon helminth infection (48), and, although most commonly associated with alternatively activated macrophages, it can also be expressed by DCs from mice infected with *Brugia malayi* (49). IRF4 is a transcription factor expressed on DCs necessary for Th2 differentiation, but not for Th1 immune responses (50). Interestingly, IRF4 also regulates the differentiation of murine mMGL2^+^ DCs, including mMGL2^+^ dermal DCs, splenic CD8α^−^ CD11b^hi^ DCs, as well as M2-macrophage polarization (51–53). Furthermore, IRF4 has been shown to bind to the IL-10 gene promoter and induces its expression in Th2 and Treg cells (54–57). Given the fact that peritoneal mMGL2^+^ CD11c^+^ cells from infected mice expressed IRF4 and that they induced the production of IL-10 by CD4^+^ T cells makes it highly likely that the production of IL-10 induction is driven by IRF4. Nevertheless, additional experiments are necessary to corroborate this hypothesis.

Our results also indicate that mMGL2^+^ CD11c^+^ cells present a higher capacity to internalize parasite molecules, as compared to mMGL2^−^ CD11c^+^ cells from control mice, explained by the increased expression of MGL or MR. This enhanced uptake could favor antigen presentation and the activation of specific CD4^+^ T cells (58). In addition, MR was recently described to interact with *F. hepatica* molecules and to mediate the partial inhibition of TLR-induced maturation of bone marrow-derived DCs (59, 60), suggesting that the parasite targets more than one CLR to evade immunity. Indeed, other CLRs, such as MR and Dectin-1, have been reported to immunomodulate Arginase-1 and PDL-2 expression and TGFβ production by macrophages in response to *F. hepatica* excretory–secretory products (61, 62).

On the other hand, mMGL2^+^ CD11c^+^ cells from infected animals suppressed the differentiation of Th1 cells induced by PAM2CSK4-stimulated DCs. These results are in agreement with previous data showing that CD11c^+^ mMGL2^+^ dermal DCs express lower levels of molecules involved in Th1-type immunity compared to CD103^+^ mMGL2^−^ dermal DCs (63). The Th1-suppressive capacity of mMGL2^+^ CD11c^+^ cells correlated with the high expression of Arg-1, PD-L1, and CCL5 (also known as RANTES) by these cells. Arg-1 has been shown to impair T cell responses by reducing the bioavailability of l-arginine and promoting l-arginine starvation (64). Moreover, suppressive DCs can upregulate Arg-1 expression (65). Murine mMGL2^+^ CD11c^+^ cells also upregulated PD-L1, which is together with PD-L2, the ligand of PD-1, an immune inhibitor receptor expressed on T cells that limits/controls cell proliferation serves to maintain immune homeostasis (66). Moreover, deficiency of PD-L1 boosts immune responses (67). PD-L1 on DCs could play a role in controlling the induction of parasite-specific immunity to allow its survival. Finally, CCL5 is a chemokine that attracts T cells eosinophils and basophils, and it recruits leukocytes to the site of infection (68). Interestingly, helminth infections are associated with high levels of CCL5, among other pro-inflammatory chemokines (26, 69), and in particular, by DCs (49). However, the pro-inflammatory function of CCL5 is inversely correlated with its extracellular levels. At low levels, RANTES serves to promote the recruitment of leukocytes to the site of inflammation, while at high levels, CCL5 stops acting as a chemokine and has direct immunostimulatory and proapoptotic activities (68). The direct function of these molecules during *F. hepatica* infection remains to be investigated.

**Figure 9:**
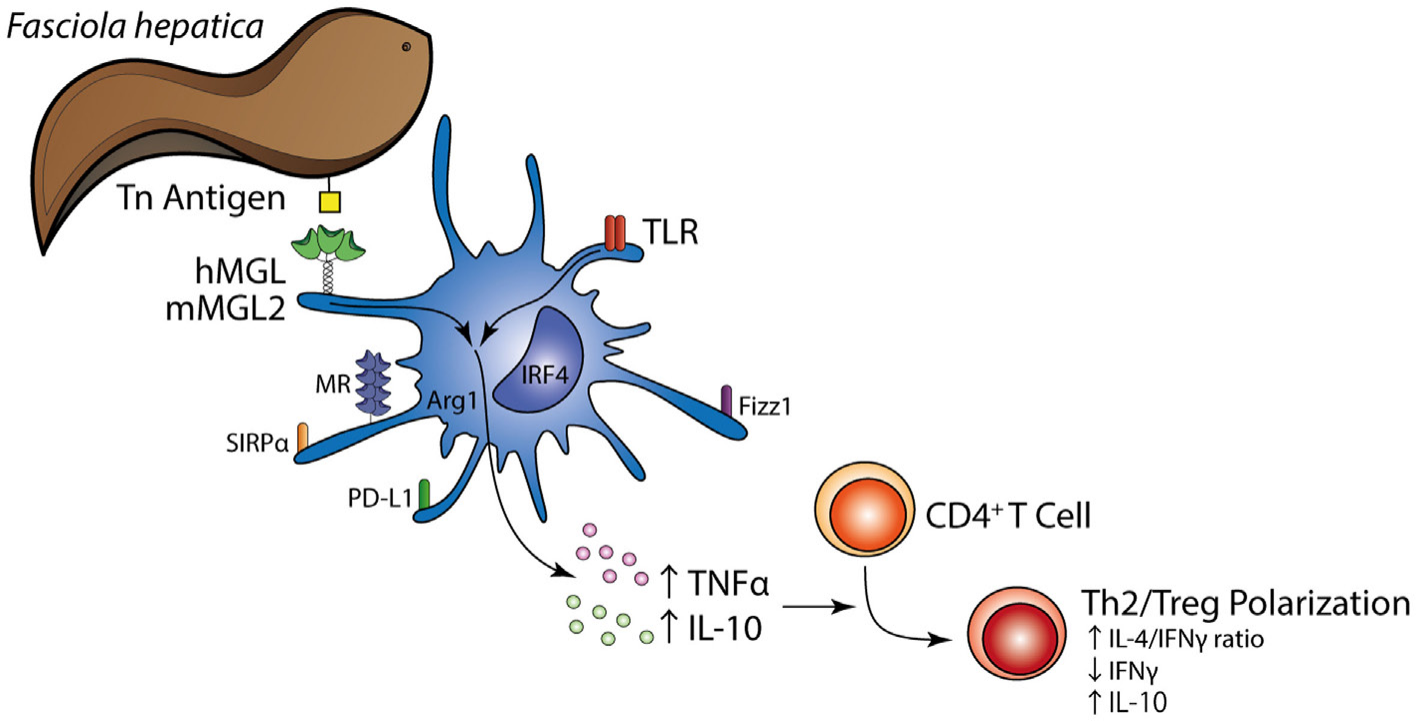
**Schematic illustration summarizing the main findings in the present study**. The Tn antigen on *F. hepatica* interacts with MGL expressed on the DC surface, triggering a regulatory program together with TLR signaling that induces enhanced expression of TNFα and IL-10 by DCs and a Th2/regulatory T cell polarization.

In conclusion, although inflammation-promoting aspects of MGL, especially for mMGL1, have been reported, considering the results presented in the study and based on the fact that MGL-induced the IL-10-mediated differentiation of Tr1 cells by DCs, we suggest that *F. hepatica* triggers anti-inflammatory properties of MGL that lead a regulation both of innate and adaptive parasite immunity (Figure 9). mMGL2^+^ CD11c^+^ cells expressing regulatory molecules (IL-10, TGFβ, PD-L1, Sirpα, Arg-1, and FIZZ1) are recruited to the peritoneum and liver of infected mice where they could expand specific Th2 and Treg cells and suppress Th1 polarization. To our knowledge, this is the first report that provides evidences about the involvement of MGL during a helminth infection and to the generation of Th2 and regulatory T cell response induced by *F. hepatica*. Moreover, this work constitutes the first report that recapitulates the *in vitro* findings on human MGL in an *in vivo* mouse model, showing that human MGL and mouse MGL2 may induce similar responses.

## Author Contributions

ER performed the experiments, analyzed data, and contributed with manuscript revision. PC contributed with experiments that involved DC function and phenotyping. SF carried out microscopy analyses. VC participated in real-time RT-PCR experiments. SV contributed with expertise involving the human MGL experiments. VN and NB participated in mouse infections and extracts preparation and detoxification. YK helped with manuscript revision. JG-V designed and supervised the experiments involving human cells and contributed to manuscript revision. TF contributed to supervision and design of all experiments shown in this paper, analyzed data, and prepared the manuscript.

## Conflict of Interest Statement

The authors declare that the research was conducted in the absence of any commercial or financial relationships that could be construed as a potential conflict of interest.
