# Supplementary material for: Fasciola hepatica Immune Regulates CD11c+ Cells by Interacting with the Macrophage Gal/GalNAc Lectin
Source: Front Immunol. 2017 Mar 15;8:264. doi: 10.3389/fimmu.2017.00264 (PMC5350155; doi:10.3389/fimmu.2017.00264)
Supplement: Supplementary file 1 [file Presentation_1.ppt]

## Slide 1
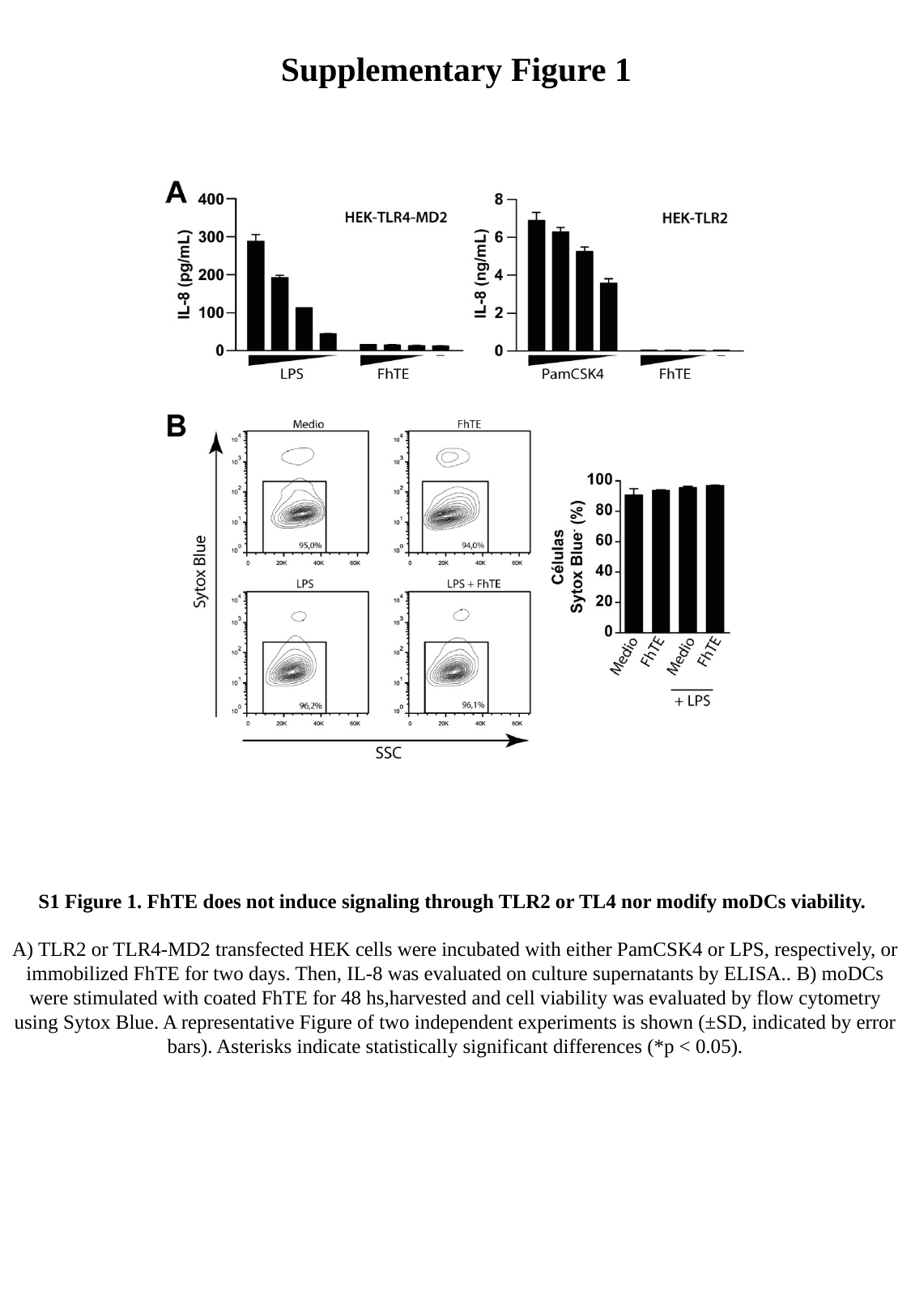

Supplementary Figure 1
S1 Figure 1. FhTE does not induce signaling through TLR2 or TL4 nor modify moDCs viability.
A) TLR2 or TLR4-MD2 transfected HEK cells were incubated with either PamCSK4 or LPS, respectively, or immobilized FhTE for two days. Then, IL-8 was evaluated on culture supernatants by ELISA.. B) moDCs were stimulated with coated FhTE for 48 hs,harvested and cell viability was evaluated by flow cytometry using Sytox Blue. A representative Figure of two independent experiments is shown (±SD, indicated by error bars). Asterisks indicate statistically significant differences (*p < 0.05).

## Slide 2
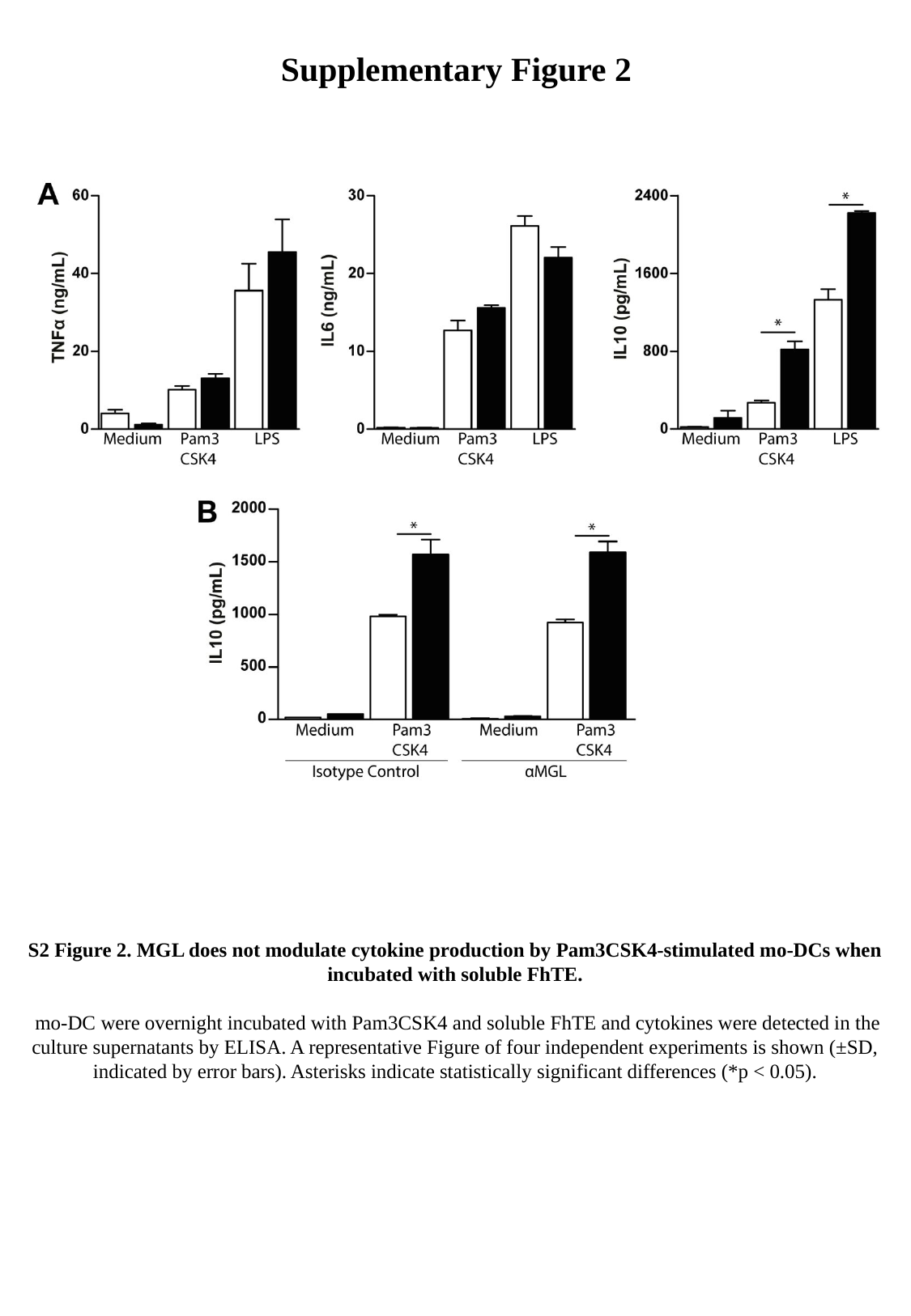

Supplementary Figure 2
S2 Figure 2. MGL does not modulate cytokine production by Pam3CSK4-stimulated mo-DCs when incubated with soluble FhTE.
 mo-DC were overnight incubated with Pam3CSK4 and soluble FhTE and cytokines were detected in the culture supernatants by ELISA. A representative Figure of four independent experiments is shown (±SD, indicated by error bars). Asterisks indicate statistically significant differences (*p < 0.05).

## Slide 3
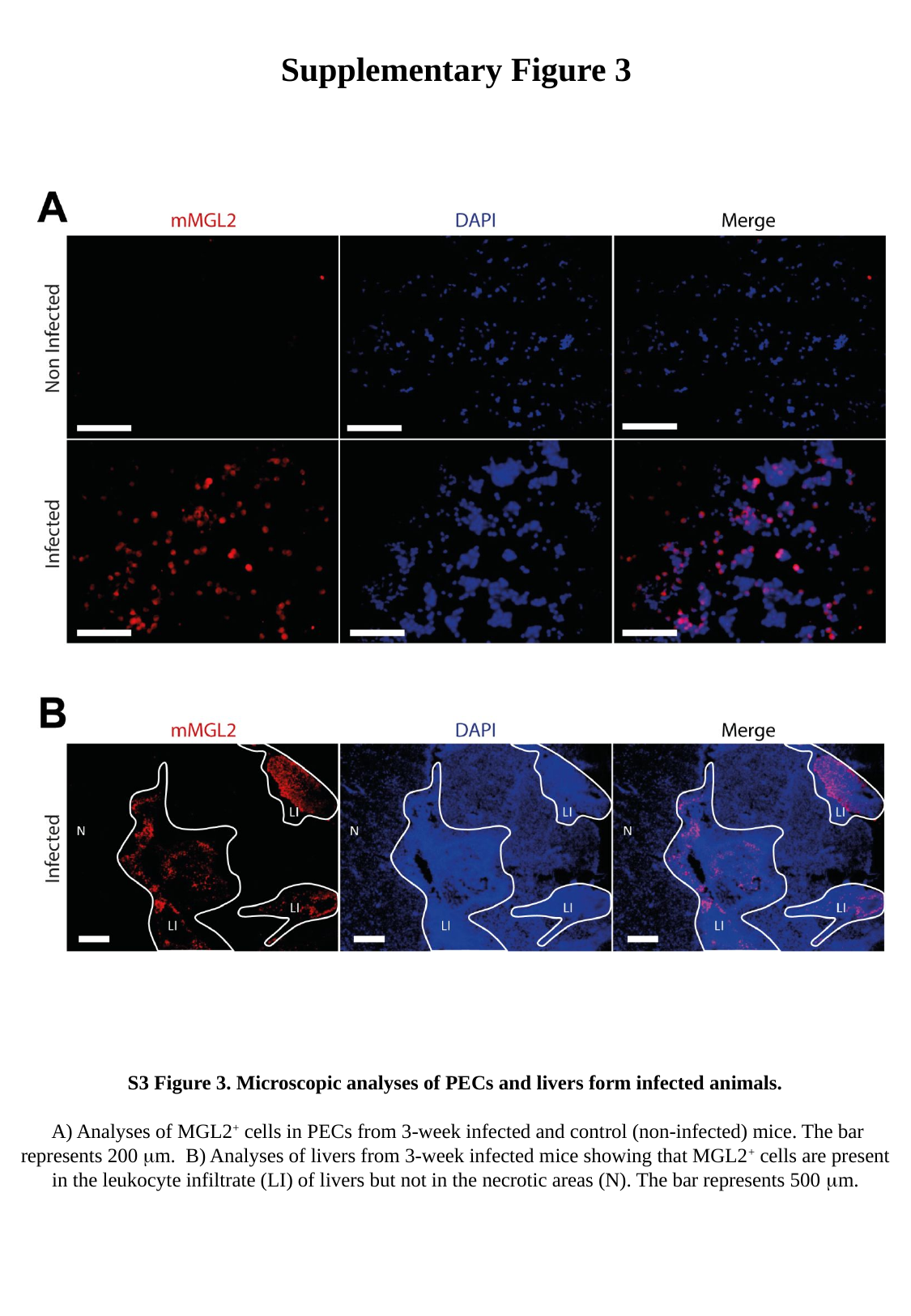

Supplementary Figure 3
S3 Figure 3. Microscopic analyses of PECs and livers form infected animals.
 A) Analyses of MGL2+ cells in PECs from 3-week infected and control (non-infected) mice. The bar represents 200 μm. B) Analyses of livers from 3-week infected mice showing that MGL2+ cells are present in the leukocyte infiltrate (LI) of livers but not in the necrotic areas (N). The bar represents 500 μm.
